# Research and Development of Cement-Based Dynamic Water Grouting Material for the CSM Construction Method

**DOI:** 10.3390/ma19061167

**Published:** 2026-03-17

**Authors:** Zhigang Yang, Fansheng Zhang, Yong Chang, Xihao Yang, Jianjian Li, Qiang Feng, Hongbo Wang, Hao Tong

**Affiliations:** 1 College of Civil Engineering and Architecture, Shandong University of Science and Technology, Qingdao 266590, China; 13325322936@163.com (Z.Y.); fansheng0228@163.com (F.Z.); 18263820556@163.com (X.Y.); fengqiang@sdust.edu.cn (Q.F.); 2 The Fifth Engineering Co., Ltd., China Railway First Engineering Group, Baoji 721013, China; changyong202602@163.com (Y.C.); 13354443231@163.com (J.L.); 3 College of Pipeline and Civil Engineering, China University of Petroleum, Qingdao 266580, China; 15079316585@163.com

**Keywords:** dynamic water environment, CSM construction method, drouting material, anti-dispersion, impermeability

## Abstract

Cutter soil mixing (CSM) is a widely adopted construction technique for forming waterproof diaphragm walls in underground engineering. However, cement slurry is prone to dispersion loss and performance degradation in moving water, making it difficult to meet engineering requirements. In this study, based on the characteristics of the CSM method in dynamic water environments, ordinary Portland cement is used as the main material, and hydroxypropyl methyl cellulose (HPMC), redispersible latex powder, polypropylene fiber and a polyether defoamer are added to improve it. The influence of each component on the performance of the new material is investigated, and a new CSM material suitable for dynamic water environments is developed. The material has good stability and suitable fluidity, controllable setting time, good anti-dispersion performance in dynamic water. The optimal mix ratio is as follows: water–cement ratio of 1; HPMC 1.4%; redispersible latex powder 3%; polypropylene fiber 0.4%; and polyether defoamer 0.8%. Field tests show that the new grouting material applied to CSM waterproof curtain construction results in a leak-free wall with excellent waterproofing performance, which verifies its engineering feasibility and provides a technical reference for the application of the CSM method in dynamic water environments.

## 1. Introduction

With the development of underground space toward more complex hydrogeological conditions, grouting reinforcement technology under dynamic water conditions has become a key problem in foundation treatment, tunnel anti-seepage and other projects. As an efficient cement–soil mixing method, the CSM method can form a large-diameter and high-strength cement–soil curtain, making it suitable for the construction of water-stop curtains in composite strata. However, in dynamic water environment, slurry is susceptible to water erosion, resulting in dispersion loss, uncontrolled setting time and deterioration of mechanical properties, which seriously affect the quality and safety of the project. The anti-dispersion and stability of traditional grouting materials under dynamic water conditions are insufficient, and it is difficult to meet the CSM method’s synergistic requirements for material fluidity, setting time and anti-scouring ability. Developing specialized grouting materials tailored for dynamic water environments is therefore an imperative research and engineering priority.

At present, scholars at home and abroad have made considerable progress in the research and development of dynamic water grouting materials, the study of slurry diffusion laws, and the optimization of construction technologies. In their research on modified cement-based grouting materials, Wu et al. [1] found that for polyacrylate latex-modified cement grouting materials subjected to dynamic water scouring, increasing the polymer–cement ratio can mitigate the deterioration of pore structure and mechanical properties. Wang et al. [2] developed a new type of water-rich grouting material with prominent plastic deformation characteristics by adding setting accelerators, alkali activators and other additives, enabling it to adapt to borehole deformation. Li et al. [3] optimized the performance of water-rich grouting materials via the response surface methodology and identified the water–cement ratio as the most critical factor affecting material performance. Wang et al. [4] prepared a new type of nano-composite cement-based dynamic water grouting material by introducing various additives into the composite material of sulphoaluminate cement and ordinary Portland cement. Wang et al. [5] developed a modified cement–clay grouting material whose scouring retention rate is 30% higher than that of cement slurry at a water flow velocity of 1.2 m/s, with a significantly reduced volume shrinkage rate. Yu et al. [6] developed a red mud-based grouting material using red mud industrial solid waste as the main raw material; this material integrates the advantages of green low-carbon characteristics and high performance and exhibits excellent injectability and diffusivity in water-rich sand layers. In the field of polymer and chemical grouting materials, Li et al. [7] synthesized a superabsorbent expandable polymer grouting material with an expansion ratio of 1:300, which effectively solves the problems of easy dilution and difficult solidification of traditional materials. Feng et al. [8] developed a new type of polyurethane water plugging material that can react normally under the condition of extensive water seepage in fractured coal and rock mass with adjustable gel time, realizing the integration of water plugging and reinforcement in one step. Song et al. [9] clarified the plugging parameters of polyurethane slurry in multi-stratum media under dynamic water conditions through experiments, providing specific technical guidance for the emergency treatment of water inrush in foundation pits.

In the research on slurry performance and mechanical characteristics, Li et al. [10] confirmed that silica fume and fibers can enhance the strength of the slurry, and redispersible polymer powder can improve the erosion resistance of the slurry. Ou et al. [11] systematically analyzed the influence of cellulose ether molecular parameters on the mechanical properties of cement slurry and found that cellulose ether increases the porosity and reduces the strength of cement slurry, with a greater reduction amplitude in compressive strength than in flexural strength. Wang et al. [12] revealed the mechanism of dynamic water scouring-induced attenuation of mechanical properties of cement grouting materials through microscopic experiments, verifying that the increase in water flow velocity leads to a significant rise in the number of micropores in set cement, as well as an obvious decrease in compressive strength and elastic modulus. Zhang et al. [13] determined the optimal mix ratio of cement–fly ash grouting materials for goafs through mix ratio tests, providing a reference for the engineering application of grouting materials with high fly ash content.

In the research on slurry diffusion laws and grouting technologies, Liu et al. [14] developed a new type of grouting material suitable for dynamic water conditions by testing the pumpability, dynamic water retention rate and other indicators of slurry with different water–cement ratios and admixture contents. Zhu et al. [15] established a dynamic water grouting model based on OpenFOAM (v1812) and found that the slurry diffusion under the action of dynamic water presents obvious asymmetry, and the water flow velocity and grouting rate have a significant impact on the diffusion distance. Gu et al. [16] and Li et al. [17] explored the influencing factors of the diffusion radius of dynamic water grouting in rock mass fractures and the rheological characteristics and diffusion mechanism of cement-based slurry with different water–cement ratios through experiments and theoretical modeling, respectively. They confirmed that the slurry viscosity has time-varying characteristics, which will be weakened with the increase in water–cement ratio. Wang et al. [18] built a visual grouting simulation system for karst straight pipelines, clarified that the dynamic water flow velocity is the external dominant factor affecting the diffusion and plugging distance of the new WIS grouting material, and proposed the action mechanism of the slurry–water velocity ratio. In terms of construction technologies, the dynamic water information-based grouting technology proposed by Yuan et al. [19] effectively improves the guidance and controllability of curtain grouting in karst water-rich tunnels through geological data update, grouting scheme optimization and on-site dynamic control. The ultra-fine cement-based grouting material (EMCG) developed by Sha et al. [20] exhibits excellent diffusivity and reinforcement effect in water-rich sand layers, and its field application has confirmed that it can effectively improve the integrity and stability of the stratum.

In addition, some scholars have developed magnetically driven cement-based grouting materials, erosion-resistant polymer grouting materials and other materials [21,22,23,24,25,26]. Some of these materials show a significant increase in retention rate under dynamic water erosion, or can harden rapidly with the strength of the consolidated body close to that of wet soil. At the same time, numerous studies have focused on the influence of grouting pressure and slurry type on dynamic water grouting diffusion [27,28,29,30], providing theoretical support for the optimization of dynamic water grouting processes.

Although the above research has achieved certain results, it has not yet formed a grouting material suitable for CSM construction under dynamic water conditions. Therefore, in this paper, ordinary Portland cement is used as the main material, and hydroxypropyl methyl cellulose, redispersible latex powder, polypropylene fiber and polyether defoamer are added for modification. The orthogonal test method is used to systematically explore and determine the optimal ratio of slurry materials. Combined with field engineering application, the aim is to solve the problems of easy dispersion and the low retention rate of ordinary Portland cement slurry in dynamic water and to provide technical support for constructing waterproof curtains in dynamic water.

## 2. Materials Selection

The dynamic water grouting material needs to have both basic performance, economy and environmental protection. Aiming at the problems of the low retention rate of common cement and cement–chemical slurry in dynamic water, poor reinforcement effect, high cost of chemical slurry and the risk of pollution, when developing new grouting materials for CSM underwater construction, ordinary Portland cement with low price, environmental protection and excellent performance is selected as the matrix. Ordinary Portland cement has fast hydration, high strength, controllable setting, wear resistance and frost resistance, and strong engineering adaptability, but it lacks anti-dispersion. Therefore, it is necessary to improve its performance by adding admixtures.

The additives include: hydroxypropyl methyl cellulose, which can improve the anti-dispersion of the slurry and improve the retention rate of dynamic water by reducing the segregation rate and increasing the viscosity; redispersible latex powder, which can enhance waterproof and aggregate bonding and improve impermeability and erosion resistance; polypropylene fiber, which can enhance the integrity and cohesion of cement structure and optimize crack resistance, impact resistance and other properties; and polyether defoamer, which can reduce bubbles and increase compactness to improve strength and durability. In addition, each component also needs to have good compatibility; that is, after the admixture is added to the slurry, it should not destroy the structure of the cement or cause adverse chemical reactions such as precipitation or decomposition, and the components should remain well coordinated.

## 3. Test Materials and Methods

### 3.1. Test Raw Materials

The P.O 42.5 ordinary Portland cement produced by Henan Mengdian Group Cement Co., Ltd., Xinxiang, China was selected for the test, and its basic performance parameters are shown in Table 1. Hydroxypropyl methyl cellulose (HPMC) is a type of mixed cellulose ether and also a polymer surfactant. The test used hydroxypropyl methyl cellulose with a viscosity of 200,000. The redispersible latex powder was produced by Shijiazhuang Chuangsheng Building Materials Technology Co., Ltd., Shijiazhuang, China and its appearance is a white powder. Polypropylene fiber was produced by Shandong Kaiwei New Material Co., Ltd., Tai’an, China and its parameters are shown in Table 2. Polypropylene fibers with this diameter and length can not only effectively fill the internal pores of the structure, but also be uniformly dispersed in the structure. Polyether defoamer is a non-ionic surfactant, which is easily soluble in water. It has not only good compatibility, but also good defoaming performance.

### 3.2. Orthogonal Test Program

In this paper, before the start of the orthogonal test, the four influencing factors of the study were examined individually. Slurry fluidity, setting time, strength and anti-dispersion performance were used as indicators to determine the optimal ratio range of the four single factors. It was concluded that the main effect of adding polyether defoamers is to eliminate bubbles in the slurry, which has little effect on the performance of the grouting material. In order to simplify the orthogonal test, the influence of its dosage was not analyzed further. When the content of polyether defoamer is 0.8%, it can not only achieve good defoaming performance, but also effectively improve the fluidity, flexural strength and compressive strength. Therefore, the content of polyether defoamer was 0.8% in the orthogonal test. Next, through the orthogonal test, the multi-factor analysis method was used to study the influence of each component on the anti-dispersion, fluidity, stability, setting time, flexural strength and compressive strength of the slurry, and to regulate the performance of the grouting material. The optimal level combination of the test was obtained, and the CSM method was applied in the dynamic water grouting project to optimize the ratio of the material to achieve the best performance. For this orthogonal experiment, four key factors affecting the properties of the slurry were selected: water–cement ratio (A), hydroxypropyl methyl cellulose (HPMC) content (B), redispersible latex powder content (C), and polypropylene fiber content (D). Three dosage levels were set for each factor, and a four-factor, three-level orthogonal test (L9 (3^4^)) was subsequently conducted. According to the results of the single-factor test, the water–cement ratio was 1, the content of HPMC was 1.4%, the content of redispersible latex powder was 2%, and the content of polypropylene fiber was 0.4%. The adjacent content was selected as the content level of the orthogonal test. The selected content level and orthogonal test scheme are shown in Table 3 and Table 4. The contents of various materials in the table are based on the quality of the ordinary Portland cement base.

## 4. Experimental Results and Analyses

The test results in Table 5 are obtained by testing the fluidity, initial setting time, final setting time, mechanical strength of different ages and retention rate under different flow velocity conditions for each group of orthogonal test schemes.

### 4.1. Stability Analysis

According to the orthogonal test scheme, the water separation rate of the material was tested. No bleeding occurred in each group of schemes, and the slurry had good stability. This is because HPMC and redispersible latex powder have good water retention, which can not only absorb free water in the slurry, but also encapsulate water molecules in the slurry, prevent the loss of water in the slurry, and prevent the segregation and stratification of the slurry. The addition of HPMC and redispersible latex powder in the dynamic water grouting material can greatly improve the stability of the slurry.

### 4.2. Analysis of Fluidity

**Figure 1:**
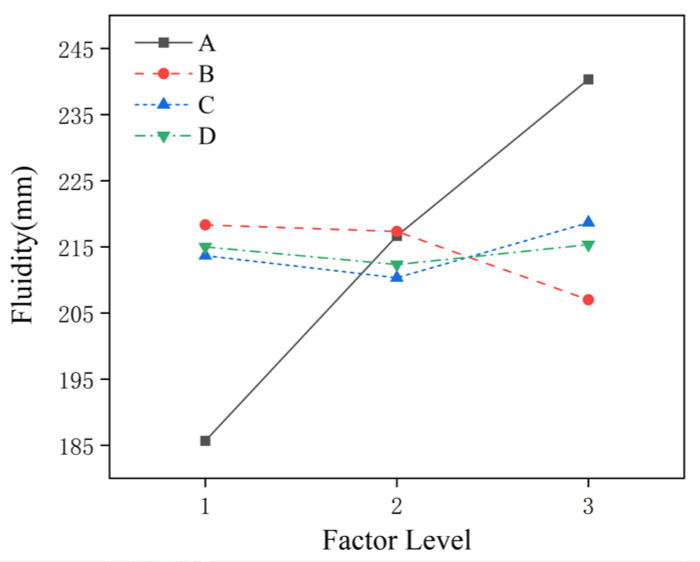
The influence of different factors on the fluidity of the slurry.

The range analysis of the test results for the fluidity in the orthogonal test scheme is shown in Figure 1. From the diagram, it can be seen that the primary and secondary relationships affecting the fluidity of the slurry are the water–cement ratio, hydroxypropyl methyl cellulose, redispersible latex powder, and polypropylene fiber. The combination ratio with better fluidity is A3B1C3D1; that is, the water–cement ratio is 1.2, HPMC is 1.3%, redispersible latex powder is 3%, and polypropylene fiber is 0.3%.

The water–cement ratio is the most important factor affecting the fluidity of the slurry. The fluidity of the slurry increases with the increase in the water–cement ratio. The increase in the water–cement ratio increases the content of free water in the slurry, thereby increasing the fluidity of the slurry. The amount of HPMC also has a significant effect on the fluidity of the slurry. HPMC can increase the viscosity of the slurry, reduce the content of free water in the slurry, and reduce the fluidity of the slurry. After the redispersible latex powder is added to the slurry, it can have a lubricating effect on the slurry and increase fluidity. Polypropylene fiber can increase the flow resistance of the slurry and have an adverse effect on fluidity.

### 4.3. Analysis of Setting Time

**Figure 2:**
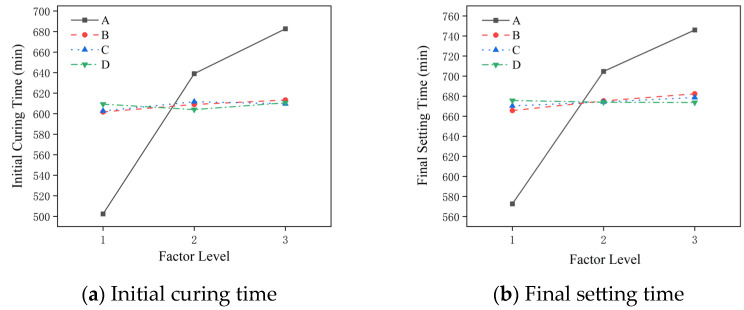
The influence of different factors on the coagulation time of the slurry.

According to the range analysis of the condensation time based on the orthogonal test results, Figure 2 is obtained. From the diagram, it can be seen that the primary and secondary relations of the factors affecting the initial setting time of the slurry are the water–cement ratio, hydroxypropyl methyl cellulose, redispersible latex powder, and polypropylene fiber. The primary and secondary factors affecting the final setting time of the slurry are the water–cement ratio, hydroxypropyl methyl cellulose, redispersible latex powder, and polypropylene fiber. The water–cement ratio is the most significant factor affecting the setting time of the slurry, and the setting time of the slurry increases with the increase in the water–cement ratio. HPMC also has an important influence on the setting time of the slurry, and the setting time of the slurry increases with the increase in HPMC content. Although the effect of HPMC content in the ranges of 1.3%, 1.4% and 1.5% on the setting time is not as significant as the water–cement ratio, through the previous single-factor test analysis, it can be concluded that the setting time of the slurry can be significantly prolonged compared with the net slurry after adding HPMC. HPMC can not only form a three-dimensional network structure in the slurry, encapsulate the particles in the slurry, and reduce the contact opportunities between the particles during the hydration process, but also increase the viscosity of the slurry and the movement resistance of the particles during the hydration process, thus prolonging the setting time of the slurry. Therefore, the setting time of the slurry can meet the construction requirements of the CSM method by selecting a reasonable water–cement ratio and HPMC content to ensure that the slurry has excellent working performance.

The setting time of the slurry in the dynamic water grouting project should not be too long. The long setting time will reduce the retention rate due to the long scouring time, which will affect the effect of grouting. For the CSM construction method, the slurry setting time should not be too short, as setting time that is too short will jam the construction machinery and affect the construction progress. Therefore, a better combination for the initial setting time is A2B2C3D1; that is, a water–cement ratio of 1; HPMC at 1.4%; redispersible latex powder at 3%; and polypropylene fiber at 0.3%. The better combination for the final setting time is A2B2C2D2; that is, a water–cement ratio of 1; HPMC at 1.4%; redispersible latex powder at 2%; and polypropylene fiber at 0.4%.

### 4.4. Strength Analysis

(1) Flexural Strength

**Figure 3:**
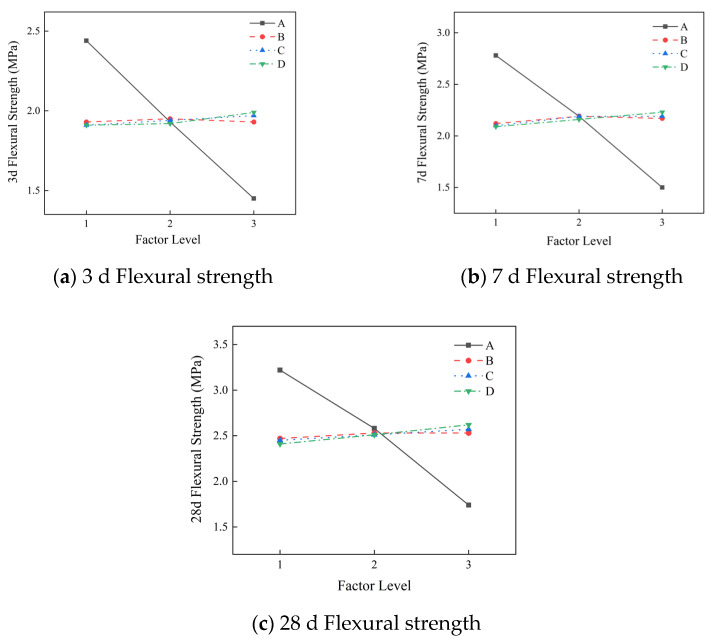
The influence of different factors on the flexural strength of the slurry.

Figure 3 is obtained by range analysis of the flexural strength of different ages (no fewer than three groups, with the average value taken) in the orthogonal test of the slurry. It can be seen from the figure that the primary and secondary factors affecting the flexural strength of the slurry at 3 d, 7 d and 28 d are the water–cement ratio, polypropylene fiber, redispersible latex powder, and hydroxypropyl methyl cellulose. The better combination is A1B2C3D, that is, a water–cement ratio 0.8, HPMC at 1.4%, redispersible latex powder at 3%, and polypropylene fiber at 0.5%.

The most important factor affecting flexural strength is the water–cement ratio, and the flexural strength of the slurry decreases with the increase in the water–cement ratio. The redispersible latex powder and polypropylene fiber also have a significant effect on the flexural strength of the slurry, and flexural strength increases with the increase in their contents. The polypropylene fiber can be uniformly dispersed in the slurry to form a network structure, which increases the traction force inside the stone body and increases the flexural strength. The redispersible latex powder can improve the viscosity and toughness of the slurry and have a favorable effect on the flexural strength. Due to the addition of a defoaming agent in the orthogonal test, the effect of bubbles on the flexural strength was reduced, so HPMC had no significant effect on the flexural strength.

(2) Compressive Strength

**Figure 4:**
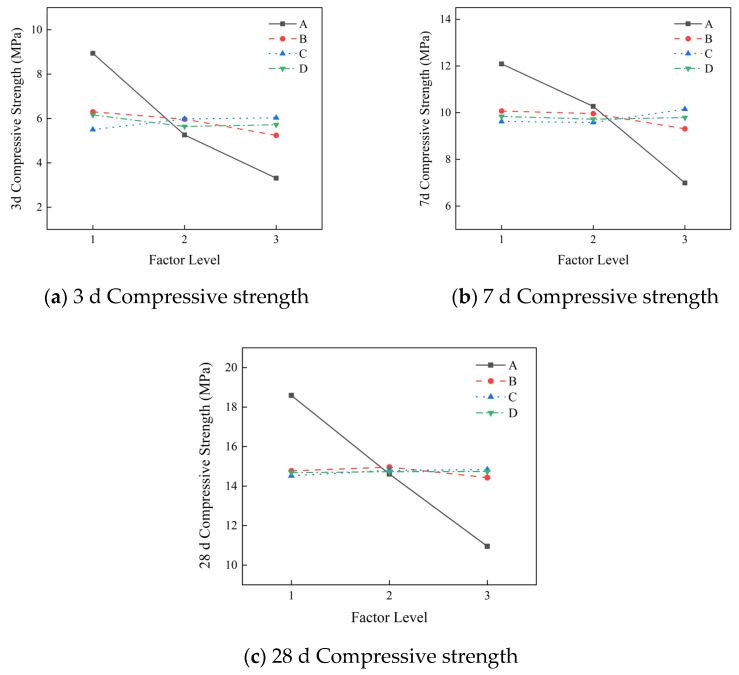
The influence of different factors on the compressive strength of the slurry.

The results of range analysis of compressive strength at different ages (no fewer than three groups, with the average value taken) are shown in Figure 4. It can be seen from the figure that the primary and secondary factors affecting the compressive strength of the slurry at 3 d, 7 d and 28 d are the water–cement ratio, hydroxypropyl methyl cellulose, redispersible latex powder, and polypropylene fiber. The better combination is A1B1C3D3, namely a water–cement ratio 0.8, HPMC at 1.3%, redispersible latex powder at 3%, and polypropylene fiber at 0.5%.

The water–cement ratio is the most significant factor affecting the compressive strength of the slurry, and the compressive strength decreases with the increase in the water–cement ratio. Although the addition of defoamer to the slurry eliminates some bubbles, it cannot completely eliminate the influence of bubbles on the compressive strength of the slurry. HPMC can increase the number of bubbles in the slurry, reduce the density of the stone body, and lead to a decrease in compressive strength. In addition, after HPMC is added to the slurry, it wraps the cement particles, which is not conducive to the development of the hydration reaction and results in decreased strength. The redispersible latex powder and polypropylene fiber have two effects on the compressive strength of the slurry: on the one hand, they can physically fill the internal pores of the structure and have a beneficial effect on the compressive strength of the slurry; on the other hand, they can increase the toughness of the stone body and adversely affect the compressive strength of the slurry.

### 4.5. Analysis of Dynamic Water Retention Rate

**Figure 5:**
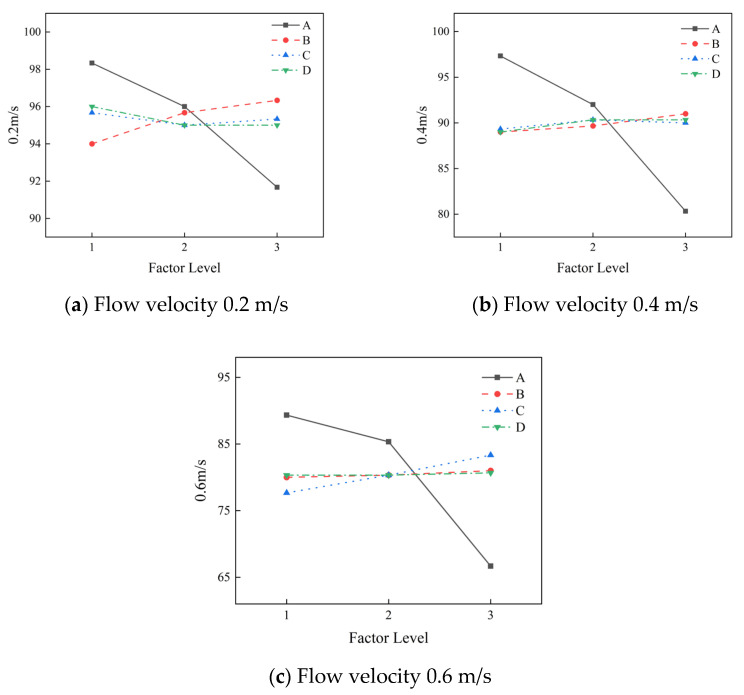
The influence of different factors on the retention rate of the slurry.

The most important performance of the grouting material is the anti-dispersion performance of the slurry in moving water. Slurry with good anti-dispersion is not easily lost under moving water and has a high retention rate. Through analysis of the data in the orthogonal test, it can be seen that the slurry also has a high retention rate in dynamic water with a flow rate of 0.6 m/s, indicating that it can resist erosion at a certain flow rate, and has good anti-dispersion performance in dynamic water. The retention rate in moving water shows a downward trend with the increase in flow velocity, which is due to the strong scouring ability of flow with large flow velocity. Range analysis of the test data was carried out, and the results for the slurry retention rate at different flow rates were obtained, as shown in Figure 5. The primary and secondary factors affecting the retention rate of the slurry at a flow rate of 0.2 m/s can be obtained from the figure: water–cement ratio, hydroxypropyl methyl cellulose, polypropylene fiber, and redispersible latex powder. The better combination is A1B3C1D1; that is, a water–cement ratio of 0.8, HPMC 1.5%, redispersible latex powder 1%, polypropylene fiber 0.3%. The primary and secondary factors affecting the retention rate of the slurry at a flow rate of 0.4 m/s are the water–cement ratio, hydroxypropyl methyl cellulose, polypropylene fiber, and redispersible latex powder. The better combination is A1B3C2D2; that is, a water–cement ratio of 0.8, HPMC 1.5%, redispersible latex powder 2%, and polypropylene fiber 0.4%. The primary and secondary factors affecting the retention rate of the slurry at a flow rate of 0.6 m/s are the water–cement ratio, redispersible latex powder, hydroxypropyl methyl cellulose, and polypropylene fiber. The better combination is A1B3C3D3; that is, a water–cement ratio of 0.8, HPMC 1.5%, redispersible latex powder 3%, and polypropylene fiber 0.5%.

The most influential factor on the slurry retention rate at different water flow rates is the water–cement ratio. The retention rate of the slurry decreases with the increase in the water–cement ratio, which means that the anti-dispersion of the slurry decreases with the increase in the water–cement ratio. The reason for this phenomenon is that with the increase in water–cement ratio, the content of free water in the slurry increases, which leads to an decrease in the viscosity of the slurry, an increase in the fluidity of the slurry, and the weakening of the resistance to water flow. On the other hand, the content of free water under different water–cement ratios is quite different. Due to the small range of HPMC content selected in the orthogonal test, the ability of HPMC to bind free water within the range of this content does not differ significantly. As the water–cement ratio increases, the effect of HPMC on increasing slurry viscosity gradually decreases, resulting in a decrease in slurry retention rate.

In the process of studying the influencing factors of the retention rate, the influence of water–cement ratio on the retention rate of the slurry was not analyzed. When the flow velocity is less than 0.6 m/s, the main factors affecting the retention rate of the slurry are HPMC and polypropylene fiber. When the water flow rate is greater than 0.6 m/s, the main factor affecting the slurry retention rate is redispersible latex powder. Under the condition of low flow rate, the addition of HPMC and polypropylene fiber can improve the retention rate of the slurry. This is because HPMC is a high-molecular polymer with functional groups that adsorb particles in the slurry. The HPMC molecular chains in the slurry can be interlaced to form a stable three-dimensional network structure, so that the particles are connected and the cohesion is increased. The viscosity of the slurry is improved, and the slurry is not easily dispersed. Polypropylene fiber can interlace into a network structure within the slurry, increasing the integrity of the structure, improving the traction force between the internal structures, and improving the anti-erosion performance of the slurry. Under high-flow rate conditions, the addition of redispersible latex powder can significantly improve the retention rate of the slurry. This is because redispersible latex powder can be uniformly dispersed in the slurry and form a mucosa. As an adhesive, it can play a reinforcing role, increase the bonding performance between the materials, and significantly improve the cohesion of the slurry, enabling it to resist the erosion of high-flow-rate water.

According to the comprehensive analysis, the CSM construction method requires grouting materials with excellent anti-dispersion performance and a high dynamic water retention rate for construction under dynamic water conditions. Additionally, the materials should possess appropriate fluidity and a controllable setting time to facilitate mechanized construction, while their flexural and compressive strengths must be sufficiently high to resist the scouring impact of water flow. Therefore, the ratio of grouting materials in the CSM method under dynamic water is determined to be a water–cement ratio of 1, HPMC1.4%, redispersible latex powder 3%, polypropylene fiber 0.4%, and polyether defoamer 0.8%.

### 4.6. Analysis of Material Advantages

Compared with ordinary cement materials, the developed dynamic water grouting material has good anti-dispersion performance and high retention rate. Compared with chemical slurry materials, the material is cheap and environmentally friendly; compared with other dynamic water grouting materials, its strength, fluidity and setting time can be adequately applied to the CSM construction method construction projects, without affecting the operation of the mechanical milling wheel, ensuring good performance of the CSM wall. The addition of HPMC improves the dispersion resistance of the material. The addition of redispersible latex powder and polypropylene fiber improves erosion resistance. In addition, polypropylene fiber also improves the crack resistance and impermeability of the material. The addition of polyether defoamer increases the strength and durability of the material.

## 5. Engineering Application

### 5.1. Engineering Situations

**Figure 6:**
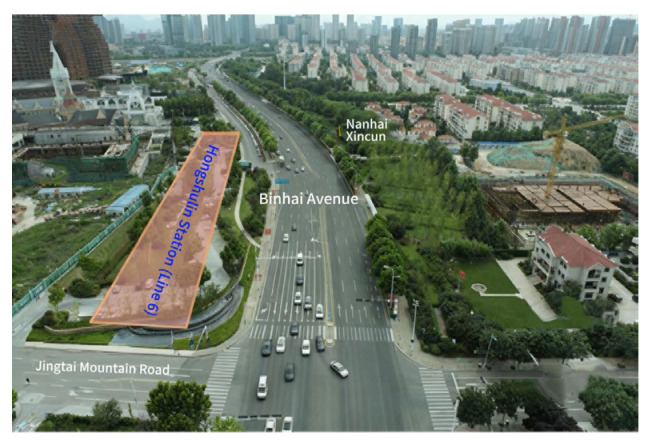
Station location distribution map.

The Mangrove Forest Station is the intermediate station of the second phase of Qingdao Metro Line 6 (Qingxi Station~Xintun Road), as shown in Figure 6. It is located in the mangrove parking lot on the west side of the intersection of Binhai Avenue and Jingtaishan Road. The terrain of the station is relatively flat, and the site elevation ranges from 3.78 to 4.97 m. The geomorphic unit is mainly a coastal shoal landform. The foundation pit of Mangrove Station is 220 m long, 20.1–23.8 m wide and 21.85–24.1 m deep. The groundwater is mainly phreatic water and bedrock fissure water, both of which are abundant. The water depth is 2.8–4.6 m. There is no confined water at the station site. The annual variation in groundwater level is 1–2 m. The groundwater level in the coastal environment of the site is significantly affected by tidal action and surface runoff recharge. The stratum is mainly composed of clay layers, sand layers and other composite strata. It has strong permeability and is connected with the water layer, forming an active groundwater flow environment. The traditional waterproof curtain construction faces problems such as groove collapse, cement slurry dilution and erosion, and foundation pit leakage.

### 5.2. Test Materials and Ratios

**Figure 7:**
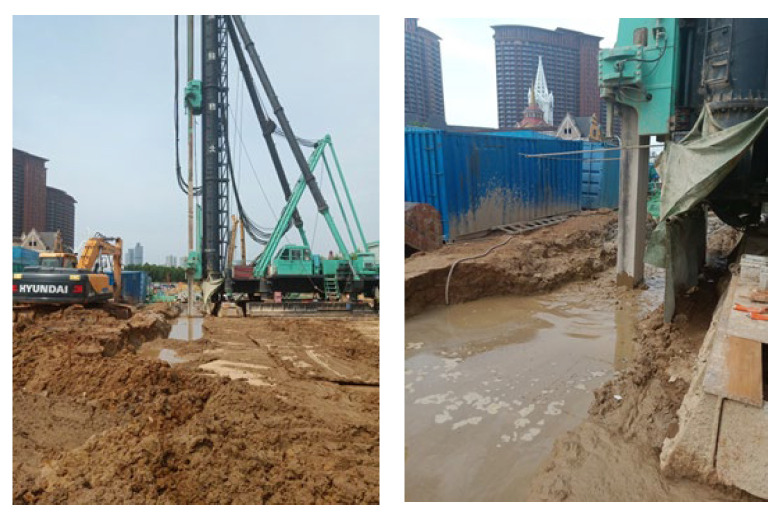
CSM wall site construction drawing.

The commonly used construction materials in CSM construction are cutting fluid and curing fluid. The selected materials and ratios should meet not only the requirements of fluidity and setting time, but also of strength and impermeability of the wall. The bentonite slurry was used as the cutting fluid, and the developed dynamic water grouting material was used as the curing fluid. The material ratio was: P.O 42.5 ordinary Portland cement with a water–cement ratio of 1, HPMC at 1.4%, redispersible latex powder at 3%, polypropylene fiber at 0.4%, and polyether defoamer at 0.8%. The cement content of the solidified liquid in the cement soil should be 20%, and the specific content can be adjusted in real time according to the on-site construction situation (Figure 7), and should not be less than 20%.

### 5.3. Wall Effect Verification

(1) Core drilling

**Figure 8:**
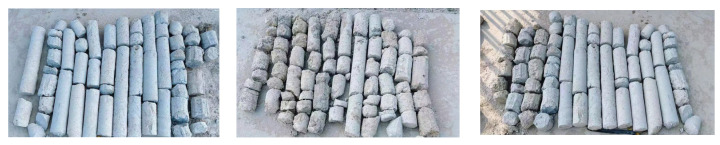
Drilling and coring results.

After 28 days of CSM wall formation, drilling and coring were carried out on the wall to test the effect of wall formation. A total of 12 core samples with a diameter of 80 mm were taken, as shown in Figure 8. The unconfined compressive strength test and permeability coefficient test were carried out on the core samples. The tests were carried out according to ‘Technical code for building foundation treatment’ JGJ79-2012 [31]. The number of test blocks should not be fewer than three, and the average value should be taken as the strength and permeability coefficient of the test. Through the experimental test, the unconfined compressive strength is greater than 1 MPa and the permeability coefficient is less than 1 × 10^−7^ cm/s, which meets the requirements of the specification, indicating that the quality of the CSM wall is good.

(2) High-definition borehole TV detection

**Figure 9:**
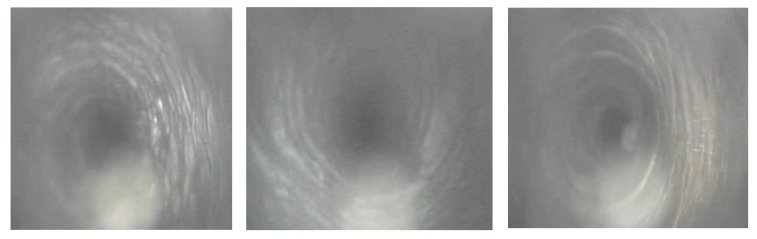
Drilling TV effect.

In order to intuitively judge the integrity of the CSM wall, high-definition borehole TV is used to observe the surface of the borehole in the wall, and to detect whether there are holes and loose defects in order to verify the wall-forming effect. The inspection using high-definition borehole TV is shown in Figure 9. As can be seen, there are no obvious defects in the wall borehole, and the quality is good.

(3) Site construction situation

**Figure 10:**
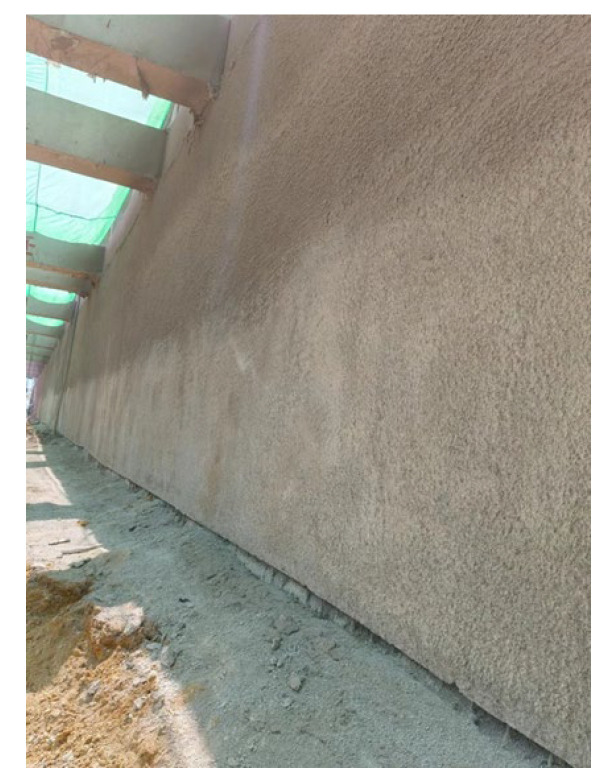
Retaining structure around the foundation pit during excavation (no leakage).

The retaining structure around the foundation pit during excavation is shown in Figure 10. It can be seen from the figure that there is no leakage on the side wall of the foundation pit. During the excavation process, there was no water gushing or sand gushing on the side wall and the bottom of the pit, and the water-stopping effect of the CSM wall was good.

Through a series of measures such as borehole coring, high-definition borehole TV detection and on-site construction observation, the wall-forming effect of the wall was evaluated. The results show that the wall forming effect is good, which verifies the reliability of the research materials mentioned above, ensures the safe construction of foundation pit engineering, and provides a more stable grouting material for CSM construction under dynamic water conditions in the future.

## 6. Conclusions and Outlook

### 6.1. Conclusions

The effects of the water–cement ratio, HPMC, redispersible latex powder and polypropylene fiber on the stability, fluidity, setting time, flexural strength and compressive strength at different ages, and retention rate at different water flow rates of dynamic underwater grouting materials were investigated by an orthogonal test. The slurry performance was dynamically regulated, and the experimental formulations under different conditions were optimized. The influence of different admixtures on the properties of solidified liquid materials was evaluated.

Through orthogonal experimental analysis, the optimal mix proportion of the grouting material for the CSM construction method under dynamic water conditions was determined as follows: a water–cement ratio of 1, a HPMC dosage of 1.4%, a redispersible polymer powder dosage of 3%, a polypropylene fiber dosage of 0.4%, and a polyether defoamer dosage of 0.8%. After optimizing the ratio through the orthogonal test, the R & D material shows good stability, suitable fluidity and controllable setting time under dynamic water conditions. The 28 d compressive strength reaches 14.93 MPa, and the retention rate in dynamic water at a flow velocity of 0.6m/s is more than 90%. The synergistic balance of anti-dispersion, construction and mechanical properties is achieved, meeting the needs of CSM dynamic water grouting project.

Based on the CSM method construction project at Mangrove Station of Qingdao Metro Line 6, engineering application was carried out. Verification of the wall-forming effect shows that the developed material provides good water-stopping performance without leakage, making it suitable for the construction of water-stop curtains using the CSM method in dynamic water environments.

The core innovation of this study is that it specifically addresses the technical challenge posed by the lack of special grouting materials for the CSM construction method in dynamic water environments, and overcomes the limitation of single-performance optimization for traditional dynamic water grouting materials. For the first time, the optimal mix ratio of a special dynamic water grouting material for the CSM construction method with clearly defined component proportions is determined. This fills the technical gap and provides a new material solution for the construction of waterproof curtains in composite strata under dynamic water conditions in underground engineering.

### 6.2. Outlook

Research on the adaptive optimization of the material for multi-scale complex dynamic water environments should be carried out. For extreme working conditions, including high-velocity dynamic water with a flow rate over 1.0 m/s, coastal tidal fluctuations, dry–wet alternation and salinized dynamic water, the mix ratio of admixtures should be adjusted or new functional admixtures introduced to enhance the material’s resistance to scouring and salt corrosion as well as its environmental adaptability.

Research on the green modification and functional upgrading of the material should be conducted. On the premise of ensuring its core performance, industrial solid wastes such as fly ash, slag and silica fume should be used to replace part of the cement, so as to reduce the material’s carbon emission and engineering cost and realize the green and low-carbon development of the grouting material.

## Figures and Tables

**Table 1:** Basic properties of cement.

Specific Surface Area (m^2^/kg)	Fineness (%)	Setting Time (min)		Flexural Strength (MPa)		Compressive Strength (MPa)	
		Initial Setting	Final Setting	3 d	28 d	3 d	28 d
377	6.4	202	263	5.5	9.8	31.3	49.6

**Table 2:** Polypropylene fiber parameters.

Diameter (µm)	Length (mm)	Density (g/cm^3^)	Tension Strength (MPa)	Elastic Modulus (MPa)	Extension at Break (%)
38	6	0.91	550	3850	28

**Table 3:** Orthogonal experimental factor level table.

Horizontal	Factors			
	Water–Cement Ratio (A)	HPMC (B)/%	Redispersible Latex Powder (C)/%	Polypropylene Fiber (D)/%
1	0.8	1.3	1	0.3
2	1	1.4	2	0.4
3	1.2	1.5	3	0.5

**Table 4:** Orthogonal experimental plan.

Test Scheme	A	B/%	C/%	D/%
1	0.8	1.3	1	0.3
2	0.8	1.4	2	0.4
3	0.8	1.5	3	0.5
4	1	1.3	2	0.5
5	1	1.4	3	0.3
6	1	1.5	1	0.4
7	1.2	1.3	3	0.4
8	1.2	1.4	1	0.5
9	1.2	1.5	2	0.3

**Table 5:** Orthogonal experimental results.

Test Scheme	Fluidity (mm)	Setting Time (min)		Flexural Strength (MPa)			Compressive Strength (MPa)			Retention Rate (%)		
		Initial Setting Time	Final Setting Time	3 d	7 d	28 d	3 d	7 d	28 d	0.2 m/s	0.4 m/s	0.6 m/s
1	190	492	561	2.38	2.61	3.02	9.39	12.27	18.41	98	95	86
2	183	503	573	2.43	2.84	3.23	9.02	12.01	18.92	98	98	89
3	184	512	584	2.51	2.89	3.40	8.42	11.99	18.44	99	99	93
4	218	639	695	1.97	2.25	2.64	5.75	10.36	14.77	94	92	85
5	225	643	711	1.94	2.19	2.56	5.91	10.87	14.93	97	91	88
6	207	635	708	1.88	2.14	2.53	4.13	9.57	14.14	97	93	83
7	247	674	741	1.45	1.49	1.76	3.77	7.59	11.13	90	80	69
8	244	681	742	1.48	1.54	1.81	2.99	7.01	11.02	92	80	64
9	230	693	755	1.41	1.47	1.66	3.17	6.37	10.71	93	81	67

## Data Availability

The original contributions presented in this study are included in the article. Further inquiries can be directed to the corresponding author.
